# Sequence analysis of *pfcrt* and *pfmdr1* genes and its association with chloroquine resistance in Southeast Indian *Plasmodium falciparum* isolates

**DOI:** 10.1016/j.gdata.2016.04.010

**Published:** 2016-04-18

**Authors:** Hiasindh Ashmi Antony, Sindhusuta Das, Subhash Chandra Parija, Sanghamitra Padhi

**Affiliations:** a Department of Microbiology, Jawaharlal Institute of Postgraduate Medical Education and Research (JIPMER), Puducherry 605006, India; b Department of Microbiology, Maharaja Krishna Chandra Gajapati Medical College (MKCG Medical College), Odisha 760004, India

**Keywords:** Drug resistance, *Plasmodium falciparum*, Chloroquine, *pfcrt*, *pfmdr1*, CQ, chloroquine, *pfcrt*, *Plasmodium falciparum* chloroquine resistance transporter, *pfmdr1*, *Plasmodium falciparum* multidrug resistance 1, SNP, single nucleotide polymorphism, K76T, lysine residue replaced by threonine at 76th position, N86Y, asparagine replaced by tyrosine at 86th position, Y184F, tyrosine replaced by phenylalanine at 184th position, AQ, amodiaquine, LD, linkage disequilibrium

## Abstract

**Background:**

Due to the widespread resistance of *Plasmodium falciparum* to chloroquine drug, artemisinin-based combination therapy (ACT) has been recommended as the first-line treatment. This study aims to evaluate the extent of chloroquine resistance in *P. falciparum* infection after the introduction of ACT. This study was carried out based on the mutation analysis in *P. falciparum* chloroquine resistant transporter (*pfcrt*) and *P. falciparum* multidrug resistance 1 (*pfmdr1*) genes. Identification of these molecular markers plays a significant role in monitoring and assessment of drug resistance as well as in designing an effective antimalarial drug policy in India.

**Methods:**

Sixty blood samples were collected from patients infected with *P. falciparum* from JIPMER, Puducherry and MKCG Medical College, Odisha. Polymerase chain reaction-restriction fragment length polymorphism was performed, targeting the point mutation of K76T in *pfcrt* and N86Y in *pfmdr1* gene. The PCR products were sequenced, genotyped and further analysed for amino acid changes in these codons.

**Results:**

The frequency of *pfcrt* mutation at 76th position was dominant for mutant T allele with 56.7% and wild type K, 43.3%. Majority of *pfmdr1* 86 allele were wild type, with N (90%) and mutant, Y (10%). Additionally, we found three haplotypes for CQ resistance, SVMNT, CVIET and CVIKT in association with the *pfcrt* gene. However, a poorly studied SNP in *pfmdr1* gene (Y184F) associated with CQ resistance showed high frequency (70%) in *P. falciparum* isolates.

**Conclusions:**

The point mutation K76T of *pfcrt* is high in *P. falciparum* suggesting a sustained high CQ resistance even after five years of the introduction of ACTs for antimalarial therapy. The present study suggests a strong association of CQ resistance with *pfcrt* T76, but not with the *pfmdr1* Y86 mutation. However, sequence analysis showed that Y184F mutation on *pfmdr1* gene was found to be associated with high resistance. Also, a new *pfcrt* haplotype ‘CVIKT’ associated with CQ resistance was found to be present in Indian strains of *P. falciparum*. The data obtained from this study helps in continuous monitoring of drug resistance in malaria and also suggests the need for careful usage of CQ in *Plasmodium vivax* malarial treatment.

## 1 Introduction

Malaria is a serious public health burden occurring mainly in the tropical and subtropical regions of the world. WHO estimated 1.2 billion people are at high risk of malaria infection globally [1]. It is a vector-borne infectious disease, caused by Apicomplexan parasite belonging to *Plasmodium* genus, which is transmitted to humans through an infected *Anopheles* mosquito during the blood meal. Of the five human-infecting malaria parasites, *Plasmodium falciparum* is the most virulent and deadliest parasite and responsible for high mortality and morbidity rate. The emanation of drug resistance in *P. falciparum* is a major threat to the malaria control and eradication program running across the globe. The first chloroquine (CQ) resistance in *P. falciparum* was reported in Southeast Asia along the Thai-Cambodian border during the late 1950s [2], [3]. In India, 50% of the malaria infections are due to *P. falciparum* and predominately in the forest, and hilly areas [4]. The first report of CQ resistance was recorded from Karbi-Anglong district, Assam in 1973 [5].

CQ kills the parasite by accumulating in the digestive vacuole and inhibiting the heme detoxification pathway [6], [7]. CQ resistance occurs due to the reduced accumulation of the drug in the digestive vacuole of the parasite. There are two molecular markers available for determining the CQ resistance: *P. falciparum* chloroquine resistance transporter (*pfcrt*) and *P. falciparum* multidrug resistance (*pfmdr1*). The *pfcrt* gene localised on chromosome 7 has 13 exons and ten putative transmembrane domains spanning the digestive vacuole of the parasite [8], [9]. *pfmdr1* gene located on chromosome 5 encodes for P-glycoprotein homologue 1 (Pgh1) protein present in the digestive vacuole of the parasite [10], [11]. Mutation in the PfCRT protein at K76T is the primary determinant for CQ resistance [8], [12], whereas N86Y mutation in PfMDR1 protein may enhance the degree of resistance [11]. Mutation in other regions of these PfCRT and PfMDR1 protein also confers resistance to CQ, but exclusively in the presence of K76T mutation.

The present study was aimed to ascertain the prevalence of *pfcrt* K76T and *pfmdr1* N86Y mutation in two communities (high and low malaria endemic regions) of India. Thus, continuous monitoring and assessment of the molecular marker of drug resistance play a significant role in determining whether resistance has faded after the cessation of drug usage. Also, it helps to map the evolutionary analysis of *pfcrt* haplotypes and to identify new mutations in the *pfcrt* and *pfmdr1* gene. In the current study, clinical isolates from two communities were collected and CQ molecular marker, *pfcrt* and *pfmdr1*, were studied and analysed to understand the degree of CQ resistance and mapping of *pfcrt* haplotypes.

## 2 Results

### 2.1 RFLP analysis

A total of 60 positive samples of *P. falciparum* were collected from JIPMER, Puducherry (n = 30) and MKCG Medical College, Odisha (n = 30) with unknown CQ sensitivity. DNA from these isolates were analysed for CQ resistance based on the presence of K76T and N86Y mutation in *pfcrt* and *pfmdr1* gene respectively. Restriction digestion of the *pfcrt* product with *Apo*I enzyme produced two fragments (128 & 136 bp) in 26 isolates showing the presence of K76 codon, which indicates sensitivity to CQ. The remaining 34 isolates were resistant to digestion conferring the presence of mutant T76 codon and CQ drug resistance (Table 1). The *pfmdr1* gene product of six samples gets digested into two fragments (253 & 350 bp) with *Afl*III enzyme, indicating the presence of mutant Y86 codon conferring CQ resistance. Rest of the isolates (n = 54) were CQ-sensitive indicating the presence of wild type N86 codon (Table 1). There was no significant association between the *pfcrt* K76T and *pfmdr1* N86Y mutation in our study population.

### 2.2 Sequence analysis

**Fig. 1:**
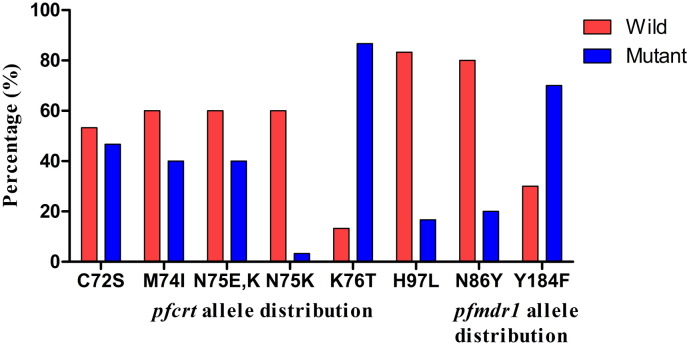
Distribution of *pfcrt* and *pfmdr1* alleles *P. falciparum* isolates collected from Puducherry and Odisha, India. K76T mutation in the *pfcrt* gene showed higher frequency associated with CQ-resistance. However, in the *pfmdr1* gene, the Y184F mutation showed a higher distribution of frequency.

The *pfcrt* and *pfmdr1* PCR amplicons were sequenced to confirm the presence of K76T and N86Y mutations. Additionally, the sequencing results were used to find out the new mutations in *pfcrt* and *pfmdr1* regions. The *pfcrt* and *pfmdr1* gene product were sequenced for a total of 30 *P. falciparum* samples (n = 15 from each centre, randomly) in both forward and reverse direction. Further, these sequences were aligned by using Clustal Omega tool, which identified six SNPs in the *pfcrt* gene and three SNPs in the *pfmdr1* gene. The DNA sequence reads of both genes were translated into amino acid codon using Expasy translate tool. Six point mutations were observed in the PfCRT amino acid sequence at 72nd, 74th, 75th, 76th and 97th position and two point mutations were recorded in PfMDR1 amino acid sequence at 86th and 184th codon position (Fig. 1). All these eight SNPs detected in both the genes were nonsynonymous substitution. One synonymous mutation in the *pfmdr1* gene, GGT to GGG coding for glycine amino acid residue at the 182nd position, was found in 20 isolates.

Overall, four haplotypes of the *pfcrt* gene (CVMNK, SVMNT, CVIET and CVIKT) were found in *P. falciparum* isolates collected from Puducherry, whereas three haplotypes (CVMNK, SVMNT and CVIET) were observed in Odisha samples. Among Puducherry isolates, SVMNT haplotype was found most frequently (n = 10), followed by CVMNK (n = 3), CVIET (n = 1) and CVIKT (n = 1) haplotypes. In Odisha isolates, CVIET haplotype was observed most frequently (n = 10), followed by SVMNT (n = 4) and CVMNK (n = 1) haplotypes. In most of *pfmdr1* gene, N86 allele was the wild type with 80%, and F184 allele was mutant with 73.33%. The *pfmdr1* gene of the Puducherry isolates frequently showed N_86_F_184_ codons (66.67%), followed by Y_86_Y_184_ (26.67%) and the wild type condon, N_86_Y_184_ (6.67%). Similarly, in Odisha isolates, N_86_F_184_ codons were more prevalent (73.33%), followed by Y_86_Y_184_ (13.33%) and wild type, N_86_Y_184_ (13.33%). Combining the mutational analysis of *pfcrt* and *pfmdr1* genes in both populations revealed the presence of 11 distinct haplotypes among the 30 Southeast Indian *P. falciparum* isolates tested in the present study (Table 2). Among the 11 haplotypes identified, H1 haplotype (S_72_V_73_M_74_N_75_T_76_H_97_N_86_G_182_F_184_) was most commonly distributed with 43.33% frequency. The H1 haplotype was more prevalent (n = 9) in Puducherry *P. falciparum* isolates and less frequent (n = 4) in Odisha population. The combined results of *pfcrt* and *pfmdr1* mutational analysis reveal the highest haplotype diversity among the *P. falciparum* isolates obtained from Odisha.

### 2.3 Assessment of genetic diversity

DNA sequence analysis of the 264 bp of *pfcrt* coding sequence show highest haplotype diversity in Odisha (Hd, 0.743) and lowest in Puducherry (Hd, 0.543) (Table 3). Also, θ_*w*_ and π estimates of Odisha *P. falciparum* isolates have the highest nucleotide diversity when compared to Puducherry isolates. Similarly, the 603 bp *pfmdr1* sequence analysis show slightly high haplotype diversity in Puducherry (Hd, 0.6) than Odisha (Hd, 0.562). The θ_*w*_ and π estimates of the nucleotide diversity show highest values in Puducherry samples than Odisha (Table 3). Also, π values are slightly higher than the θ_*w*_ values in both genes, suggesting a larger number of intermediate frequency mutations in both the population. The neutrality tests show no statistically significant results for all the three tests conducted to indicate any divergence from neutrality in both *pfcrt* and *pfmdr1* population samples. These data signifies that both population *P. falciparum* isolates are evolving under the neutral model of molecular evolution and no involvement of selection pressure in both the *pfcrt* and *pfmdr1* genes.

**Fig. 2:**
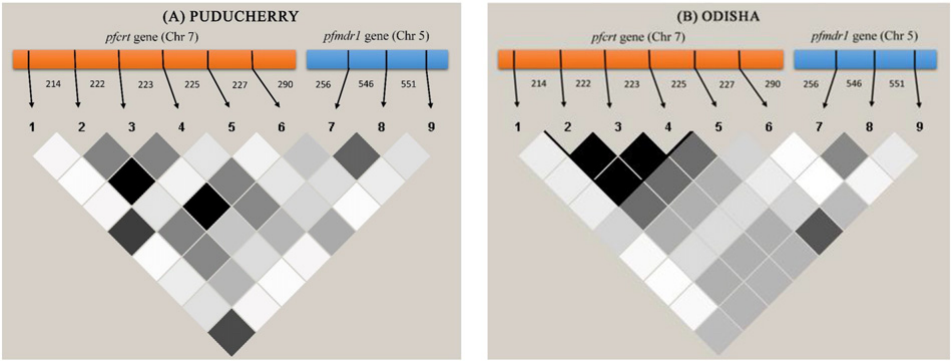
Linkage disequilibrium plot between the SNPs pairs of *pfcrt* and *pfmdr1* gene in Indian ***P.****falciparum*. The intergenic and intragenic association between the genes were illustrated using the LD plot for isolates collected from (a) Puducherry and (b) Odisha regions. The strength of LD between the SNPs is determined by the association of statistical significance by calculating the r^2^ values and represented by the darkness of the boxes.

To determine the role of natural selection in the *pfmdr1* gene, the dN and dS values were estimated for each population independently, and Z-test was performed for codon-based natural selection test (Table 3). The P value of the Z-test for natural selection was significantly higher than the 5% level of significance (data not shown), suggesting no role of evolutionary natural selection in the *pfmdr1* gene in samples of both population and it confirms with Tajima's *D* test of neutrality findings. Furthermore, LD was determined by estimating r^2^ values between all possible pairs of SNPs present in both *pfcrt* and *pfmdr1* genes to study their genetic association (Fig. 2). Statistically significant intragenic associations were detected between SNP pairs in the *pfcrt* gene, but no intergenic association found between the *pfcrt* and *pfmdr1* genes.

## 3 Discussion

The advancement in the molecular techniques is extremely useful for the detection of drug resistance in malarial parasites and plays an immense role in the epidemiological survey as well as in regular updating of the antimalarial drug policy regimes. The CQ resistance in *P. falciparum* has been associated with *pfcrt* and *pfmdr1* genes [13], [14]. However, the mutation in the *pfcrt* gene at K76T position has been considered as the primary determinant for CQ resistance [8], [12]. On the other hand, *pfmdr1* gene mutation has poor correlation with CQ resistance [11], [15]. Thus, tracking mutation in these candidate genes and finding new mutations and its association, could help us in the better understanding of how the genetic change in genes could alter the phenotypic characters, and also how genetic recombination evolve in response to drugs used for therapy [16].

This is a hospital-based prevalence study performed to monitor the CQ resistance based on the detection of the point mutation in the *pfcrt* and *pfmdr1* genes by PCR-RFLP method. In the present study, the prevalence of *pfcrt* K76T mutation was 40% and 73.33% in Puducherry and Odisha population respectively. Similarly, the N86Y mutation in the *pfmdr1* gene was found to be 13.33% and 6.67% by PCR-RFLP in Puducherry and Odisha population respectively. In some studies, the N86Y mutation was associated with CQ resistance [17], [18], [19], whereas, in other studies this association was not found [20], [21], [22], [23], [24]. In the present study population, there was no association of N86Y mutation of the *pfmdr1* gene with CQ resistance and this non-association was in agreement with previous findings [16], [20], [25], [26]. However, the prevalence of N86Y mutation was high in other Indian *P. falciparum* isolates [16], [27], [28].

The frequency of CVIET haplotype of the *pfcrt* gene was dominant in Odisha *P. falciparum* isolates and correlates with earlier reports from Odisha [16], [27]. Furthermore, the SVMNT haplotype of the *pfcrt* gene was seen with high frequency in Puducherry. The use of Amodiaquine (AQ) drug, reported to select the SVMNT haplotype in *P. falciparum* isolates [29], [30], but in Puducherry, in spite of no usage of AQ, this haplotype was found to be highly frequent. The prevalence of SVMNT haplotype of the *pfcrt* gene in Indian *P. falciparum* isolates could be due to the ongoing migration of SVMNT from Papua New Guinea to Southeast Asia and India [31], [32]. Thus, either the usage of AQ as single or in combination therapy should be avoided in future for malaria treatment in Puducherry.

Interestingly, a new haplotype CVIKT (triple mutant) of the *pfcrt* gene has been observed in Puducherry. This haplotype was first reported from Indonesia, Papua New Guinea (Indonesian Papua) with less frequency [33]. Hence, this will represent the first report of the CVIKT haplotype from Indian population, and it could be due to the migration from Papua New Guinea, like similar kind of migration observed in the case of SVMNT haplotype. Furthermore, a nonsynonymous mutation H97L in the *pfcrt* gene was observed with appreciable frequency in *P. falciparum* isolates obtained from Odisha than in Puducherry. Previously, the H97L mutation in *pfcrt* gene has been reported in CQ resistance isolates of *P. falciparum* [8], [34], [35], including the report from Odisha [27].

When the N86Y mutation of the *pfmdr1* present along with K76T mutation of the *pfcrt* gene has been argued to associate with the CQ resistance in *P. falciparum* isolates [13], [36]. Additionally, in India, statistically significant LD between these two mutations have been reported [27]. In the present study, no significant LD between the K76T mutation in the *pfcrt* and N86Y mutation in the *pfmdr1* gene was observed. However, the mutation in the 72nd, 74th and 75th position of the *pfcrt* gene showed significant LD association. Further, the results of non-association of K76T and N86Y mutation correlates with previous findings from India [16]. Although these mutations have no direct role in CQ resistance, studies have shown high LD association of 72nd, 74th and 75th mutation in the *pfcrt* gene [16], [27].

Similarly, Y184F mutation in the *pfmdr1* gene was associated with CQ resistance [13], and studies have shown its negative correlation to CQ resistance [19]. However, a previous study from Odisha has reported the high prevalence of N86Y mutation and low prevalence of Y184F mutation [27]. The Y184F mutation is associated with Artemisinin Lumefantrine (AL) resistance and N_86_F_184_ genotype in eastern Sudan have shown the increase in treatment failure to AL in *P. falciparum* isolates [37], [38]. However, in India, *P. falciparum* isolates with N_86_F_184_ genotype were sensitive to AL therapy [39]. Further, the presence of synonymous mutation, G182G, in the *pfdmr1* gene was a remarkable finding, since this mutation was present in other Indian field isolates and cultured isolates indicating the presence of this mutation since a long time in India [16]. Though this mutation does not change the amino acid, it might involve in altering transport kinetics since it is present near to the 184 amino acid residue [40].

In Odisha, the high number of haplotypes combining the *pfcrt* and *pfmdr1* genes was due to the high malaria transmission rate, resulting in high haplotype diversity. Similar findings of high haplotype diversity has been observed in *pfcrt*-*pfmdr1* genes in Odisha [16], along with microsatellite variation in and around the *pfcrt* gene [41]. Thus, whole sequence analysis of *pfcrt* and *pfmdr1* gene with larger sample size covering wider population is needed to understand clearly the molecular evolution pattern in both *pfcrt* and *pfmdr1* of *P. falciparum* isolates in India.

In the present study, the π values of nucleotide diversity were higher for *pfmdr1* gene and lower for the *pfcrt* gene in Puducherry isolates than Odisha. The finding of high nucleotide diversity for the *pfmdr1* gene in Puducherry (low endemic region) than Odisha (high endemic region) is interesting, and contrast to the general perspective of *P. falciparum* [42]. Similar findings of high nucleotide diversity were reported in another Indian state, Gujarat (low endemic region), in comparison to high malaria endemic regions of Odisha and Assam [16], [43]. The haplotype and nucleotide diversity of *pfmdr1* gene were higher in Puducherry population than Odisha, which could be due to the comparatively larger number of haplotypes. Furthermore, linkage disequilibrium between N86Y and Y184F mutation in Odisha isolates show statistically weaker association than the Puducherry isolates. Though Puducherry is considered as less endemic to *P. falciparum* malaria, genetic diversity was higher with less LD significance than Odisha isolates with less genetic diversity and weaker LD association. Large-scale genetic population studies are required to compare the LD association of low and high endemic regions of *P. falciparum* samples.

*P. vivax* malarial infection is prevalent in many parts of the world [1]. Chloroquine is the treatment of drug choice for *P. vivax* infection which kills the asexual stages of the parasite in the blood, along with primaquine that kills the hypnozoite stages of the parasite in the liver. In Papua New Guinea and Indonesia, resistance to chloroquine treatment in *P. vivax* infection was first emerged in the late 1980s [44]. Since then chloroquine resistance has been documented in many regions of the world [45], [46]. WHO recommends the use of ACT in areas where chloroquine resistance has been reported for *P. vivax* infection [1]. Hence, chloroquine should be used carefully for *P. vivax* treatment to prevent/delay the drug resistance. Several drug resistant molecular markers have been identified for the detection of drug resistance in *P. vivax* [47], [48], [49]. These markers provide valuable information in *P. vivax* treatment since in vitro culturing of *P. vivax* is difficult. Thus, drug resistant molecular marker helps in the early detection and continuous monitoring of drug resistance, which aid in effective drug policy regimes.

## 4 Conclusion

The survey of *pfcrt* and *pfmdr1* gene fragments associated with CQ resistance study in high and low malaria endemic areas of India benefit us in understanding the molecular evolutionary changes in *P. falciparum* isolates. This study has limitations due to the fewer *P. falciparum* isolates from Puducherry region due to their low endemicity and less number of DNA sequencing results in both regions. Despite its drawbacks, the results obtained from this study has its significance in unravelling the genetic diversity pattern and genetic associations between the mutations in *pfcrt* and *pfmdr1* genes of *P. falciparum* collected from low and high endemic regions of India. Thus, large-scale molecular epidemiological studies on antimalarial drug resistance markers are needed to identify the previously existing and new mutation in the particular genes that contribute to the drug resistance in malaria. All this molecular epidemiological data are in turn helpful in regular revisal of antimalarial drug policies.

## 5 Materials and methods

### 5.1 Ethical clearance

This study was reviewed and approved by JIPMER Scientific Advisory Committee and Institute Ethics Committee for sample collection in JIPMER, Puducherry. Similarly, Institutional Ethics Committee approval was also obtained for sample collection in MKCG Medical College, Odisha.

### 5.2 Sample collection & DNA extraction

Totally sixty *P. falciparum* positive blood samples collected from two tertiary care hospitals, JIPMER, Puducherry (low endemic region) and MKCG Medical College, Odisha (high endemic region), constitute the samples for this study. Routine blood samples received for malaria investigation in Parasitology Section were screened by examination of thin and thick smears using Giemsa staining and rapid diagnostic test (RDT). The samples positive for *P. falciparum* by microscopy and RDT were included in the study. Artemisinin combination therapy was provided for treatment of malaria in both the centres based on National Drug Policy of India [4].

*P. falciparum* genomic DNA was extracted from 200 μl of EDTA-blood using Qiagen Blood DNA Mini kit (Qiagen, Hilden, Germany) following manufacture's protocol, and stored at − 20 °C until further use.

### 5.3 PCR for pfcrt and pfmdr1

The K76T and N86Y mutation in the *pfcrt* and *pfmdr1* gene respectively, which are the primary determinants of markers for CQ resistance, were targeted using PCR (Agilent SureCycler 8800, CA, USA). A 264 bp *pfcrt* gene product, corresponding to 32 to 119 amino acid residues, which contain K76T mutation was amplified using the primer pair 5′-GGCTCACGTTTAGGTGGA-3′ and 5′-TGAATTTCCCTTTTTATTTCCAAA-3′, as described earlier [50]. Similarly, the *pfmdr1* gene containing the N86Y mutation in the extreme 5′ end of the gene was amplified by the primer set 5′-ATGGGTAAAGAGCAGAAAGA-3′ and 5′-AACGCAAGTAATACATAAAGTCA-3′ to obtain a 603 bp gene fragment, corresponding to 1 to 201 amino acid residues [50].

### 5.4 Restriction digestion with *Apo*I and *Afl*III

The final PCR amplified product of *pfcrt* and *pfmdr1* gene was enzymatically digested with *Apo*I and *Afl*III (New England Biolabs, UK) respectively. Digestion of the *pfcrt* PCR product into two fragments (128 & 136 bp) with *Apo*I enzyme confers that the isolate was sensitive to CQ with CVMNK haplotype, whereas, the K76 T mutation was resistant to digestion [50]. On the other hand, when the *pfmdr1* gene product was digested with *Afl*III enzyme, it cleaved into two fragments, i.e., 253 and 350 bp, and these isolates were considered as CQ-resistant with mutant Y allele at 86th position. However, CQ-sensitive isolates were resistant to restriction digestion [50]. Further, the digested fragments were resolved by electrophoresis on 1.8% agarose gel stained with ethidium bromide and visualised using gel documentation system (Biorad, USA).

### 5.5 Genotyping of pfcrt and pfmdr1

The undigested PCR products of *pfcrt* and *pfmdr1* genes were chosen randomly (15 from each centre) for sequencing to determine the haplotypes of CQ resistance in *pfcrt* gene, based on the mutation in 72–76 codons and to find novel single nucleotide polymorphisms (SNPs) in these genes. The PCR products were gel purified with FavorPrep PCR/Gel purification kit (Favorgen, Taiwan) using manufacture's protocol. Sequencing was performed using ABI 3730XL automated DNA sequencer in both forward and reverse directions (2 × coverage) at Scigenom Labs, Cochin, India. DNA sequence reads were aligned using BioEdit 7.2 software [51] and analysed using Clustal Omega tool [52]. Haplotypes of these isolates were determined based on SNPs present in these gene fragments by comparing the multiple sequence alignments with reference to the CQ-sensitive *P. falciparum* 3D7 strain. The sequence reads were deposited in GenBank with accession no. KU376443-KU376472 and KU493989-KU494018.

### 5.6 Statistical analysis

Data analysis for RFLP study was performed by using SPSS version 16 (SPSS Inc., Chicago IL) [53]. The statistical association between the point mutations and the independent variable were analysed using Chi-square test and a p-value ≤ 0.05 was considered statistically significant. The aligned *pfcrt* and *pfmdr1* coding sequences were used to determine the number of haplotypes and haplotype diversity (Hd) [54]. Also, two measures of nucleotide diversities, θ_*w*_ and π [55], [56], were estimated separately for each sample population. These parameters were calculated by using the computer program DnaSP version 5.10 [57]. Furthermore, the frequency of non-synonymous substitutions per non-synonymous site (dN) and the frequency of synonymous substitutions per synonymous site (dS) were determined for the two population, and a Z-test was performed to calculate the p-value using the MEGA, version 6 program [58]. To resolve whether there is any association between nine SNPs in two population (present in both *pfcrt* and *pfmdr1* genes), both inter and intragenic linkage disequilibrium (LD) were determined by calculating the r^2^ values for each possible pair-wise SNP, using the program Haploview [59].

## Competing interests

The authors declare that they have no competing interests.

## Figures and Tables

**Table 1:** Detection of CQ resistance in Puducherry and Odisha *P. falciparum* isolates using RFLP analysis. CQ-resistance was mainly attributed to K76T and N86Y mutation in the *pfcrt* and *pfmdr1* gene respectively. CQ-resistant parasites were higher in Odisha whereas almost equal in Puducherry regions even after the withdrawal of CQ.

	No of samples (n)	*pfcrt*-K76T		*pfmdr1*-N86Y	
Sensitive	Resistant	Sensitive	Resistant
Puducherry	30	18 (60%)	12 (40%)	26 (86.67%)	4 (13.33%)
Odisha	30	8 (26.67%)	22 (73.33%)	28 (93.33%)	2 (6.67%)
Total	60	26 (43.33%)	34 (56.67%)	54 (90%)	6 (10%)

**Table 2:** Details of haplotypes present from Puducherry and Odisha region isolates, India. The number of haplotypes were obtained by combining the mutation present in both the *pfcrt* and *pfmdr1* gene of Southeast Indian *P. falciparum* isolates. H1 haplotype showed high frequency present mainly in Puducherry region.

Haplotype ID	Haplotype	No of isolates (n)	Frequency of haplotypes (%)
H1	S_72_V_73_M_74_N_75_T_76_H_97_N_86_G_182_F_184_	13	43.33
H2	S_72_V_73_M_74_N_75_T_76_H_97_Y_86_G_182_Y_184_	1	3.33
H3	C_72_V_73_I_74_E_75_T_76_H_97_N_86_G_182_F_184_	3	10.00
H4	C_72_V_73_I_74_E_75_T_76_L_97_N_86_G_182_F_184_	4	13.33
H5	C_72_V_73_I_74_E_75_T_76_H_97_N_86_G_182_Y_184_	1	3.33
H6	C_72_V_73_I_74_E_75_T_76_H_97_Y_86_G_182_Y_184_	2	6.67
H7	C_72_V_73_I_74_E_75_T_76_L_97_Y_86_G_182_Y_184_	1	3.33
H8	C_72_V_73_I_74_K_75_T_76_H_97_Y_86_G_182_Y_184_	1	3.33
H9	C_72_V_73_M_74_N_75_K_76_H_97_N_86_G_182_Y_184_	2	6.67
H10	C_72_V_73_M_74_N_75_K_76_H_97_N_86_G_182_F_184_	1	3.33
H11	C_72_V_73_M_74_N_75_K_76_H_97_Y_86_G_182_Y_184_	1	3.33

**Table 3:** Details of genetic diversity of *pfcrt* and *pfmdr1* genes in *P. falciparum* isolates collected from Puducherry and Odisha. The genetic parameters and tests of neutrality were calculated using DnaSP program. The high haplotype and nucleotide diversity were observed in the *pfcrt* sequence analysis from Odisha isolates, which is due to the high malaria transmission rate. However, the Puducherry isolates showed high haplotype and nucleotide diversity in the *pfmdr1* analysis.

Gene		*pfcrt*		*pfmdr1*	
Population		Puducherry	Odisha	Puducherry	Odisha
No of isolates (n)		15	15	15	15
SNPs (n)		6	6	3	3
No of haplotypes (n)		4	3	4	5
Haplotype diversity (Hd)		0.543	0.743	0.6	0.562
Nucleotide diversity	θ	0.00699	0.00699	0.00153	0.00153
π	0.00599	0.00909	0.00234	0.0018

Tests of neutrality					
Tajima's D		− 0.50145	1.05243	1.56977	0.52578
Fu and Li′s D*		− 0.01484	0.63222	1.05657	1.05657
Fu and Li′s F*		− 0.16644	0.85232	1.35864	1.04652
Tajima's D (NonSyn/Syn) ratio		na	na	0.871	0.29732
